# Fabrication of Promising Antimicrobial Aloe Vera/PVA Electrospun Nanofibers for Protective Clothing

**DOI:** 10.3390/ma13173884

**Published:** 2020-09-02

**Authors:** Haleema Khanzada, Abdul Salam, Muhammad Bilal Qadir, Duy-Nam Phan, Tufail Hassan, Muhammad Usman Munir, Khalid Pasha, Nafees Hassan, Muhammad Qamar Khan, Ick Soo Kim

**Affiliations:** 1 Nanotechnology Research Lab, Department of Textile and Clothing, Faculty of Engineering and Technology, National Textile University Karachi Campus, Karachi 74900, Pakistan; haleema0119@gmail.com (H.K.); ab.salam@ntu.edu.pk (A.S.); hassan.tufail@ntu.edu.pk (T.H.); drpasha@ntu.edu.pk (K.P.); nafeeshassan@ntu.edu.pk (N.H.); 2 Department of Textile Engineering, Faculty of Engineering and Technology, National Textile University, Faisalabad 37610, Pakistan; bilal.qadir@ntu.edu.pk; 3 School of Textile-Leather & Fashion, Hanoi University of Science and Technology, Hanoi 10000, Vietnam; duynamphan@gmail.com; 4 Department of Material Engineering, Kaunas University of Technology, 44249 Kaunas, Lithuania; Muhammad.munir@ktu.edu; 5 Nanofusion Technology Research Lab, Institute of Fiber Engineering, Faculty of Textile and Sciences, Ueda Campus, Shinshu University, Nagano 386-8567, Japan

**Keywords:** Aloe Vera/PVA, nanofibers, antimicrobial, protective clothing

## Abstract

In the present condition of COVID-19, the demand for antimicrobial products such as face masks and surgical gowns has increased. Because of this increasing demand, there is a need to conduct a study on the development of antimicrobial material. Therefore, this study was conducted on the development of Aloe Vera and Polyvinyl Alcohol (AV/PVA) electrospun nanofibers. Four different fibers were developed by varying the concentrations of Aloe vera (0.5%, 1.5%, 2.5%, and 3%) while maintaining the concentration of PVA constant. The developed samples were subjected to different characterization techniques such as SEM, FTIR, XRD, TGA, and ICP studies. After that, the antimicrobial activity of the developed Aloe Vera/PVA electrospun nanofibers was checked against Gram-positive (*Staphylococcus aureus*) bacteria and Gram-negative (*Escherichia coli*) bacteria. The developed nanofibers had high profile antibacterial activity against both bacteria, but showed excellent results against *S. aureus* bacteria as compared with *E. coli*. These nanofibers have potential applications in the development of surgical gowns, gloves, etc.

## 1. Introduction

Considering the current situation due to the pandemic of COVID-19, people feel insecure about the products they are using in their daily life. Therefore, the demand for antimicrobial products is increasing day-by-day. In such a situation, nanotechnology can play a vital role in the development of antimicrobial products [1,2]. Nanotechnology is a very broad field and it can be further classified into nanoparticles [3], nanorods [4,5], nanodisks [6], nanowire [7], nanofibers [8], and nanotubes [9]. Keeping in view the current situation of COVID-19, nanofibers can play an important role in the development of antimicrobial products [10,11]. These nanofibers can be produced using different techniques such as drawing technique [12], template synthesis technique [13], phase separation technique [14], self-assembly technique [15], and electrospinning technique [16]. These nanofibers can also be used for antimicrobial products such as surgical gowns, face masks, etc. [17,18,19,20,21,22,23,24]. Several researchers have researched and developed different electrospun nanofibers for antimicrobial activity. These nanofibers were loaded with different types of materials such as nanoparticles, drugs, herbs extract, etc. Kalwar et al. conducted their study on the manufacturing of silver loaded cellulose acetate nanofibers using the electrospinning technique. They tested the antibacterial activity of these nanofibers against *Escherichia coli* and *Staphylococcus aureus* bacteria by allowing 18 h contact of nanofibers with bacteria. By using 1% Ag loaded nanofibers, a 14.4 mm zone of inhibition was observed against *S. aureus* and 16 mm against *E. coli* [25]. A similar study was conducted by Ahir et al., they loaded copper nanoparticles with electrospun nanofibers. First, poly-d and PEO, l-lactide were electrospun, and then copper nanoparticles were loaded. When copper loaded nanofibers were tested for the antibacterial activity, the results revealed that, after 48 h, these nanofibers showed 41% reduction of *P. aeruginose* and 50% reduction of *S. aureus* bacteria [26]. P. Gopinath developed CuO-ZnO loaded polyvinyl alcohol-based electrospun nanofibers and checked the antimicrobial activity against *E. coli* and *S. aureus* bacteria. They found that 450 μg/mL concentration of CuO-ZnO loaded electrospun nanofibers showed inhibited growth against *E. coli* bacteria, whereas 300 μg/mL concentration CuO-ZnO loaded electrospun nanofibers showed inhibited growth against *S. aureus* bacteria [27]. Different studies have been conducted on the development of antibacterial nanofibers by loading different drugs with electrospun nanofibers. Lui et al. synthesized poly (lactic-co-glycolic acid)/alginate electrospun nanofibers, and then the ciprofloxacin drug was loaded with these electrospun nanofibers. They found that by using the broth microdilution method, and determined 0.125 μg/mL MIC value of ciprofloxacin against *S. aureus* bacteria [28]. Han et al. determined a 99.99% decrease of bacteria (*S. aureus*) by using nisin/cellulose acetate electrospun nanofibers [29]. Sadri et al. developed PEO/chitosan electrospun nanofibers. These fibers were loaded with two types of thyme essential oils, i.e., narrow leaves and broad leaves oil. The antibacterial activity of the fibers was tested against *S. aureus* and pseudomonas aeruginosa bacteria. For nanofibers loaded with broad leaves oils, the inhibition zone was shown to be 10 mm against *P. aeruginosa* and 19 mm against *S. aureus*, whereas for nanofibers loaded with narrow leaves oil, the zone of inhibition was show to be 8 mm against *Pseudomonas aeruginosa* and 15 mm against *S. aureus* bacteria. They concluded their study by saying that oils of broader leaves show higher antibacterial activity as compare with oils of narrow leaves [30]. Aloe Vera is a medicinal plant and it has properties such as wound healing, antioxidant, antiviral, antibacterial, antidiabetic [31,32]. Aloe Vera possesses two main components, i.e., glucomannan and acemannan. These two components are responsible for the antiviral, tissue regeneration, and antibacterial effect [33]. Many researcher have used Aloe Vera in their studies for tissue engineering applications [34,35,36,37,38,39]. It has also been mentioned in the literature that Aloe Vera possessed excellent antibacterial activity against Gram-negative bacteria [40]. Numerous studies have been done on the development of Aloe Vera based scaffolds using the electrospinning technique. Mary et al. conducted their study on the development of Aloe Vera loaded polycaprolactone (PCL) electrospun matrices. They studied the wettability and degradation of Aloe Vera in PCL matrices. Their results showed that blended matrices degraded faster than PCL matrices and hydrophilicity of mats depended on the blending of Aloe Vera, i.e., hydrophilicity increased with the blending of Aloe Vera with PCL [41]. Similarly, Suganya et al. conducted their study on the development of Aloe Vera/PCL based nanofibrous scaffolds. They found that PCL with 10% Aloe Vera concentration showed enhanced hydrophilic characteristics, thinner fiber diameter, and excellent tensile strength (6.28 MPa) with a modulus of 16.11 MPa which made these nanofibers suitable for skin tissue engineering applications [42]. Sirdha et al. developed PCL electrospun nanomembrane for anticancer activity. For this purpose, they incorporated Aloe Vera/curcumin with PCL electrospun nanofibers. The prepared membranes were tested for lung cancer and breast cancer. They found 15% more cytotoxicity exhibited by PCL membranes, if 5% curcumin and 1% Aloe Vera were loaded with PLC nanofibers membrane [43]. Abdullah et al. incorporated Aloe Vera gel with Polyvinyl Alcohol (PVA) electrospun nanofibers and found that the morphology of an Aloe Vera/PVA blend was excellent as compare with pure PVA nanofibers. They found that the size of blended nanofibers was less than that of the pure nanofibers [44]. Jegina et al. developed Aloe Vera gel loaded PVA electrospun nanofibers mats by using the roller spinning technique. They loaded three different concentrations of Aloe Vera gel and found that a variation in the concentration of Aloe Vera gel effected electrical conductivity, viscosity of spinning solution, diameter of nanofibers, and mechanical properties of nanofibers. This study concluded that as the concentration of Aloe Vera gel increased, the diameter of the nanofibers decreased. They also found that mechanical properties of nanofibers mats were enhanced with the concentration of Aloe Vera gel [44]. Fatemah et al. checked the release of Aloe Vera gel from Aloe Vera (AV)/PVA electrospun nanofibers pads. They found that Aloe Vera or Aloe Vera/PVA electrospun nanofibers could be utilized in wound care as an Aloe Vera release system. At least 60 percent of the Aloe Vera was released in the first hour in a phosphate buffer solution, and about 90 percent of the Aloe Vera was released in 2–4 h, depending on the diameter of the nanofibers [45].

A lot of research has been conducted on the loading of Aloe Vera gel with different polymers for different applications, however, very little literature has been found on the loading of Aloe Vera gel with Polyvinyl Alcohol (PVA) for rapid antibacterial applications. The authors have been the first to perform antibacterial activity of AV/PVA nanofibers. Therefore, there is a need to further explore this area of research. This study focused on the development of Aloe Vera/PVA electrospun nanofibers by using the electrospinning technique. Four different concentrations of Aloe Vera gel were used for the development of electrospun nanofibers. The developed nanofibers were tested for the antimicrobial activity against *E. coli* and *S. aureus* bacteria.

## 2. Materials and Method

### 2.1. Materials

Polyvinyl Alcohol (PVA) (MW: 85,000–124,000 and 87–89% hydrolyzed) was purchased from the Sigma-Aldrich Corporation (St. Louis, MO, USA), Aloe Vera gel was extracted from Pakistani Aloe Vera plant and glutaraldehyde (GA, 50% in aqueous solution) was provided from MP Biomedical (Tokyo, Japan). Deionized water was used.

### 2.2. Preparation of Aloe Vera and Polyvinyl Alcohol (AV/PVA) Nanofibers

**Figure 1:**
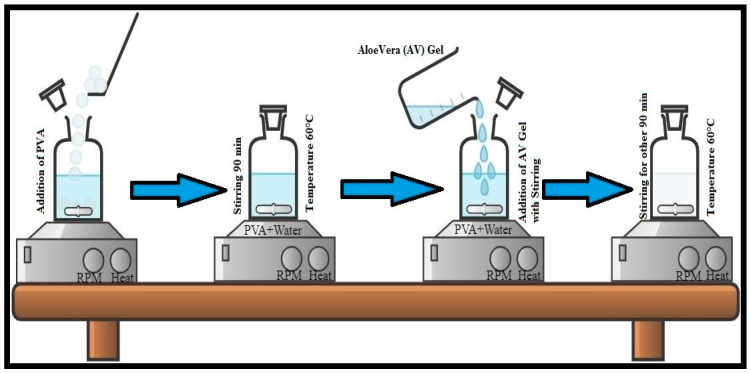
Flow diagram of Aloe Vera and Polyvinyl Alcohol (AV/PVA) solution formation process.

Four different solutions were formed by varying the concentration of Aleo vera gel, i.e., 0.5%, 1%, 2.5%, and 3%. A certain amount of distilled water was taken in the reagent bottle, then, 10% (W/V) PVA powder was added slowly to this water and stirred at 450 rpm. The temperature was slowly raised, up to 60 °C, and then stirred for about 90 min, until a clear solution was obtained. Then, 0.5% of Aleo vera (AV) gel was added slowly to this solution and stirred for about 90 min, at 500 rpm, while maintaining the temperature at 60 °C. After some time, a clear solution of AV/PVA of certain viscosity was obtained. Similar steps were followed for the formation of the other three solutions with different concentrations of Aleo vera gel. The flow diagram of the solution formation process is shown in Figure 1.

**Figure 2:**
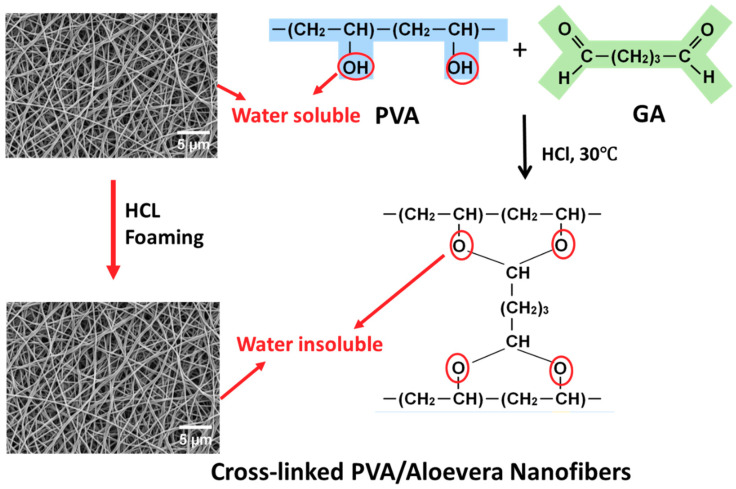
Crosslinking of PVA/Aloe Vera nanofibers.

For the production of AV/PVA electrospun nanofibers, a syringe was filled with the prepared polymer solution and attached to an electrospinning machine, and a voltage of 17 kV was applied, while maintaining a distance of 20 cm between the collector and the tip of the syringe. As the voltage was applied, the AV/PVA fibers started to be generated and were collected on the collector of the electrospinning machine. The resultant nanofibers were cross linked by the hydrochloric acid (HCL) foaming of AV/PVA/GA at 30 °C, for 60 s. In this reaction, GA and HCl acted as a chemical crosslinking agent and a catalyst, respectively, as shown in Figure 2.

### 2.3. Characterizations

Prepared electrospun nanofibers were subjected to certain characterization techniques to test the certain characteristics of the prepared electrospun nanofibers. To check the surface morphology of the electrospun nanofibers, nanofibers were analyzed by using a scanning electron microscope (SEM) JSM-5300, JEOL Ltd., Tokyo, Japan. The XRD analysis of AV/PVA electrospun nanofibers was performed at 25 °C with nanofiber samples using a Rotaflex RT300 mA (Rigaku manufacturer, Osaka, Japan) and nickel-filtered Cu. Ka radiation was used for measurements, along with an angular angle of 5 ≤ 2θ ≤ 50°. To test the chemical interactions, the analysis of the prepared electrospun nanofibers were studied by FTIR spectra (IR Prestige-21 by Shinmadzu, Nagano, Japan). The water contact angle measurements were done using a contact angle meter (Digidrop, GBX, Whitestone Way, France). Five different specimens of each sample were tested and investigated. Inductively coupled plasma atomic emission spectroscopy (ICP-AES, Shimadzu ICPS-1000 IV, Shimadzu, Kyoto, Japan) was implemented to determine AV release in solutions, for up to 72 h.

### 2.4. Evaluation of Antimicrobial Efficiency

The antimicrobial activity of all the developed samples was analyzed by using the agar diffusion test (qualitative method). For this purpose, the AATCC-147 standard method was used, and a triplication process was adopted. The AV/PVA electrospun nanofibers were tested against Gram-negative bacteria (*E. coli*) and Gram-positive bacteria (*S. aureus*). Both bacterial strains were incubated overnight at a temperature of 37 °C. Then, 45 μL of a 10^5^ dilution of stain solution was spread on sterilizing agar plates. Samples were placed transversely on the agar surface to ensure intimate contact. Then, the growth was examined, and the inhibition zone was calculated by using Equation (1) [46] and with the use of Image J Software (version 1.49). For one test specimen, 4 different reading were taken by measuring T1, T2, T3, and T4 in mm from different places on the test specimen. Then, T was calculated by taking the average of the 4 measurements. After measuring T, the zone of inhibition “W” was calculated using the following formula:

(1) $$W=\frac{\left(T-D\right)}{2}$$

where W is the width of the zone of inhibition, T is the total diameter of the test specimen and clear zone in mm, and D is the diameter of the test specimen in mm.

## 3. Results and Discussion

### 3.1. Surface Morphology of AV/PVA Electrospun Nanofibers

**Figure 3:**
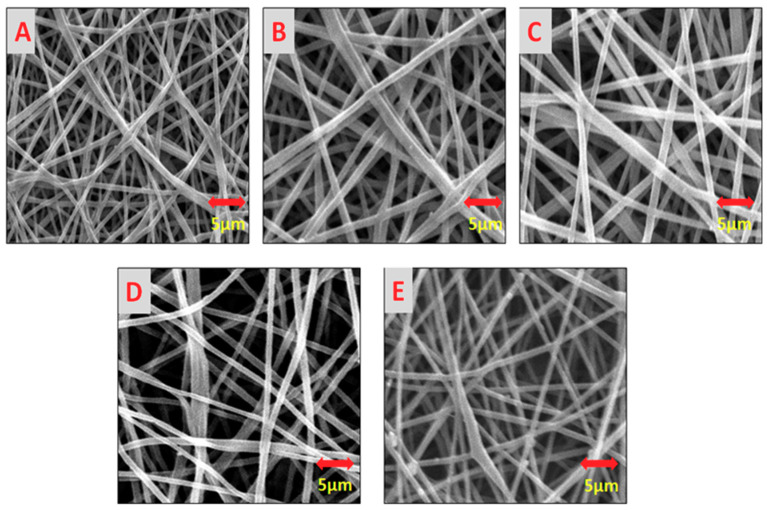
Surface morphology of the developed electrospun nanofibers. (**A**) Pure PVA nanofibers; (**B**) 0.5% AV/PVA nanofibers; (**C**) 1.5% AV/PVA nanofibers; (**D**) 2.5% AV/PVA nanofibers; (**E**) 3% AV/PVA nanofibers.

All the developed samples with Aloe Vera concentration (0.5%, 1.5%, 2.5%, and 3%) and neat PVA nanofibers were analyzed by SEM for surface morphology analysis. Five images from different places of a same sample were investigated and it was confirmed that all the fibers were smooth and there was no formation of beads that took place, as mentioned in Figure 3.

**Figure 4:**
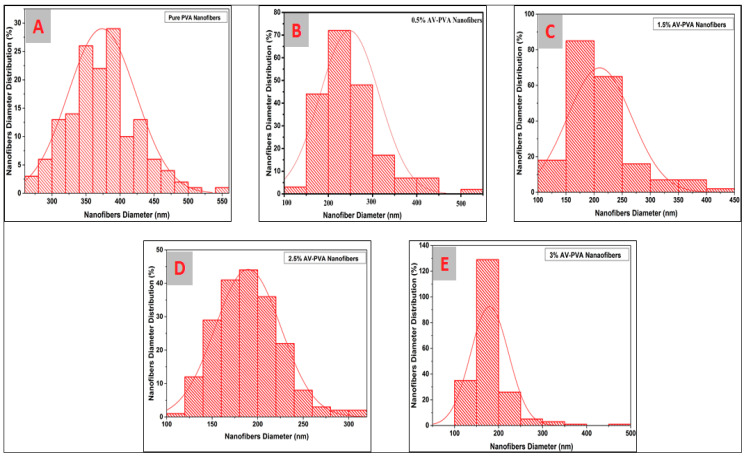
Diameter distribution of the developed electrospun nanofibers. (**A**) Pure PVA; (**B**) 0.5% AV/PVA; (**C**) 1.5% AV/PVA; (**D**) 2.5% AV/PVA; (**E**) 3% AV/PVA.

To analyze the diameter of the prepared electrospun nanofibers, ImageJ software was used. A total of 200 readings were taken from each sample to calculate the average diameter of electrospun nanofibers. Figure 4A shows the histogram of PVA electrospun nanofibers. The X-axis represents the diameter of nanofibers, whereas the Y-axis represents the diameter distribution of electrospun nanofibers. The diameter of pure PVA electrospun nanofibers falls in the range of 373.4 with a standard deviation of 0.049. In Figure 4B, 251.51 nm average diameter is observed in the case of 0.5% AV-PVA electrospun nanofibers with a standard deviation value of 0.04789 nm. Figure 4C indicates the histogram of 1.5% AV-PVA electrospun nanofibers. In this case, the average diameter falls in the range of 209.75 nm with a standard deviation value of 0.05708 nm. Figure 4D indicates a histogram of 2.5% AV-PVA electrospun nanofibers and, in this case, the average diameter falls in the range of 189.4 nm with a standard deviation of 0.03612 nm. Figure 4E indicates the histogram of 3% AV-PVA electrospun nanofibers with an average diameter of 179.59 nm having a standard deviation value of 0.04312 nm. From histograms of all the samples, it can be seen that as the concentration of the Aloe Vera gel increases the diameter of the prepared nanofibers decreases significantly. The possible reason for this behavior could be, as the concentration of Aloe Vera increases, the electrostatic forces and columbic repulsion between the molecules increase due to the presence of H groups in both PVA and Aloe Vera gel, resulting in more compactness of the molecules [47]. Hence, the diameter of the electrospun nanofibers decreases with an increase in the concentration of Aloe Vera gel. Similar results were also obtained in a previous study [45].

### 3.2. FTIR Analysis of AV/PVA Electrospun Nanofibers

**Figure 5:**
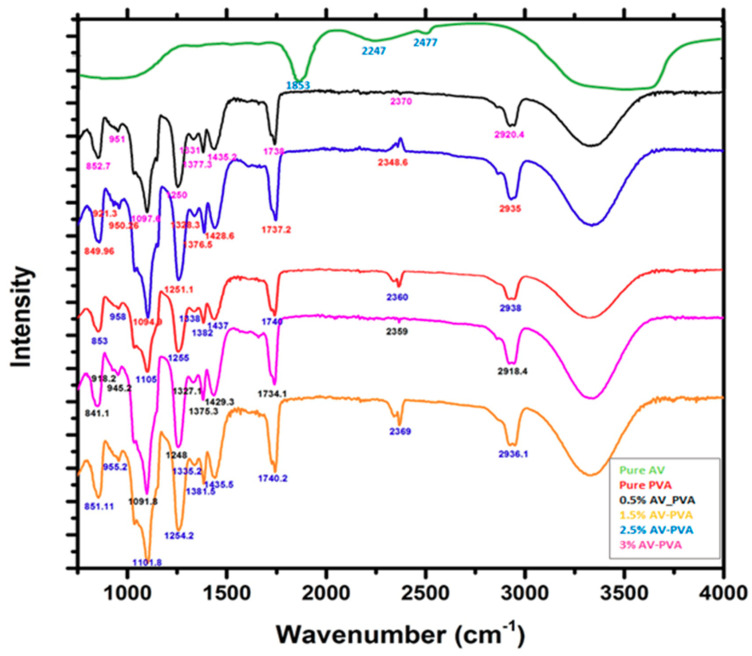
FTIR spectra of pure PVA, 0.5%, 1.5%, 2.5%, and 3% AV/PVA electrospun nanofibers.

Figure 5 indicates the FTIR spectra of pure PVA, 0.5%, 1.5%, 2.5%, and 3% Aleo vera PVA blended electrospun nanofibers. In Figure 5, the wavenumber is shown on the X-axis, and its range varies from 1000 cm^−1^ to 4000 cm^−1^. Pure Aloe Vera sperctrum shows four main peaks. The peak at 1853 cm^−1^ corresponds to the amid group. The characteristic peak appears at 2247 cm^−1^, and 2477 cm^−1^ corresponds to the corboxylic functional group. However in the pure Aloe Vera spectra, the broader peaks in the range 3000–3600 cm^−1^ correspond to the presence of the –OH functional group. All other spectra shown in the figure are almost similar to each other, with a slight shifting of peaks. In all spectra, there is a broader peak that appears in the range 3300–3500 cm^−1^, which represents the phenolic functional groups, especially anthraquinones. The peaks that appear in the range 2900–3000 cm^−1^ are due to the C–H stretching, such peaks correspond to points 2938, 2920.4, 2936.1, 2335, and 2918.4 cm^−1^ in the case of pure PVA, 0.5%, 1.5%, 2.5%, and 3% AV/PVA electrospun nanofibers, respectively. In the spectra, peaks in the range 1030–1150 cm^−1^ are due to C-O stretching. Such peaks appear at points 1105, 1097, 1101.8, 1094.9, and 1091.8 cm^−1^ in the case of pure PVA, 0.5%, 1.5%, 2.5%, and 3% AV/PVA electrospun nanofibers, respectively. Due to C=O stretching, the peaks appear at points 1740, 1738, 1740.2, 1737, and 1734.1 cm^−1^ for pure PVA, 0.5%, 1.5%, 2.5%, and 3% AV/PVA electrospun nanofibers, respectively. C=C stretching in alion and vinyl ether components are shown in the range 1400–1500 cm^−1^ and correspond to points 1437, 1435.2, 1435.5, 1428.6, and 1429.3 cm^−1^ for pure PVA, 0.5%, 1.5%, 2.5%, and 3% AV/PVA electrospun nanofibers, respectively [48,49]. The peaks in the range 1310–1390 cm^−1^ for all spectra are due to –OH bending in the phenolic group. The peaks appear between 790 and 840 cm^−1^ and correspond to C=C bending in the alkene group for all the spectra. From all the spectra, it can be concluded that there is no chemical interaction that takes place between Aloe Vera and PVA because no new peaks appear in the spectra. In the spectra, the broader peaks in the range 3300–3500 cm^−1^ indicate that the quantity of OH^−1^ groups increased with an increase in the quantity of Aloe Vera, as a result, the moisture-absorbing capacity of the material increased [45].

### 3.3. XRD Analysis AV/PVA Electrospun Nanofibers

**Figure 6:**
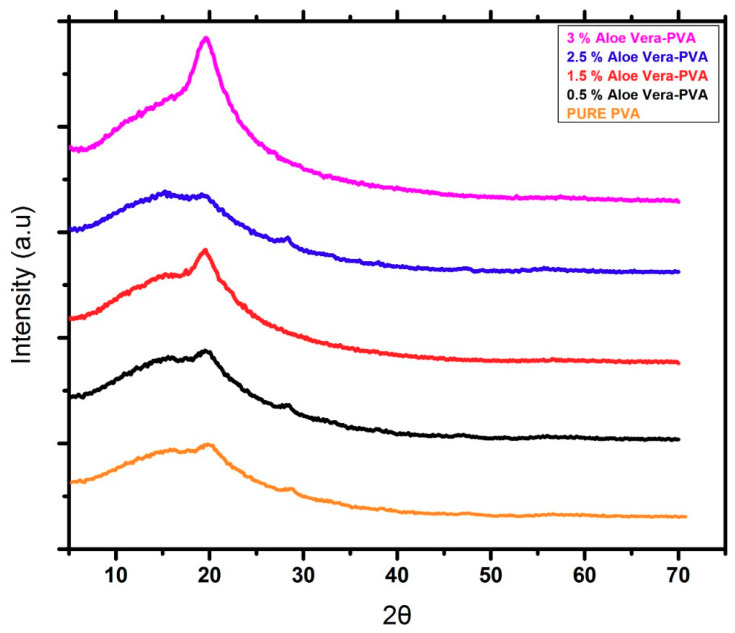
XRD spectra of pure PVA, 0.5%, 1.5%, 2.5%, and 3% AV/PVA electrospun nanofibers.

**Figure 7:**
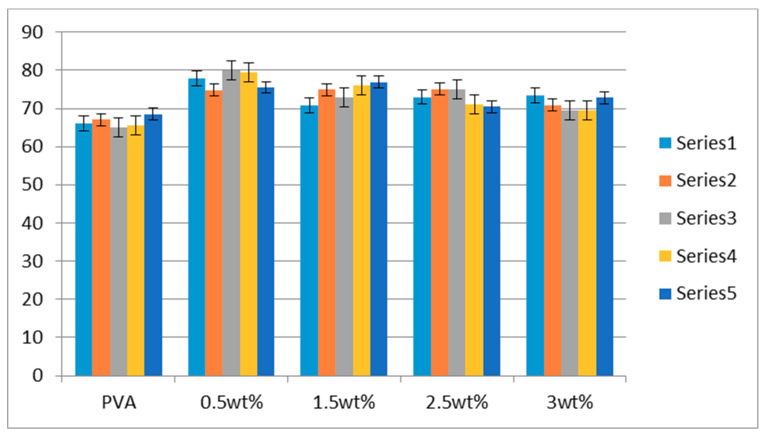
Hydrophilic behavior of all resultant samples.

Figure 6 shows the X-ray diffraction spectra of 0.5% AV/PVA, 1.5% AV/PVA, 2.5% AV/PVA, and 3% AV/PVA electrospun nanofibers. The material was tested on an analytical diffractometer at a working current and voltage of 45 mA and 45 KV, respectively. It can be seen in Figure 7 that the peaks appear at 20°. It can also be observed that as the blend ratio increases, the peaks broadening increase, which indicates that the amorphous region of the material increases. The sharp peak was obtained in the case of 0.5% AV/PVA electrospun nanofibers. However, the highest peak broadening occurred in the case of 3% AV/PVA electrospun nanofibers, which indicated the highest amount of amorphous region [45].

### 3.4. Water Contact Angle Analysis

In order to investigate the hydrophilic behavior of the PVA and AV/PVA nanofibers, water contact analysis was done, as mentioned in the Figure 7. It was confirmed that crosslinking of the PVA and AV/PVA nanofibers was done successfully, and all samples showed the hydrophilic behavior. Statistical analysis confirmed that neat PVA nanofibers showed hydrophilic behavior at 66.5° ± 2° but 0.5 wt% AV/PVA showed the higher crystalline structure, but low hydrophilic behavior as compared with the PVA, but 3 wt% as neat PVA in hydrophilic behavior.

### 3.5. Release Analysis by ICP

**Figure 8:**
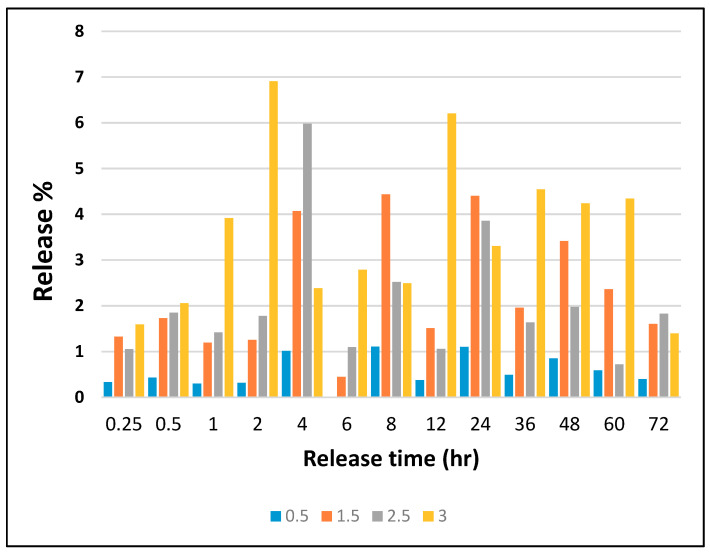
Release analysis by ICP study.

In order to investigate the release behavior of loaded AV in the AV/PVA nanofibers, as shown in Figure 8, nanofibers were immersed in 50 mL of deionized water, for up to 72 h. The ICP analysis was subsequently done on these solutions to investigate the amount (%) of AV loaded in the AV/PVA nanofibers. It was confirmed that the release behavior was very poor which was in favor of protective clothing such as masks because these products are used for the external environment of the human body. The maximum release of AV was in the sample of 3 wt% AV/PVA nanofibers which was 6.8% of total loaded AV. It is a very low amount and poor release behavior, but all samples have a good release profile as required for protective clothing.

### 3.6. Degradation Analysis by TGA Study

**Figure 9:**
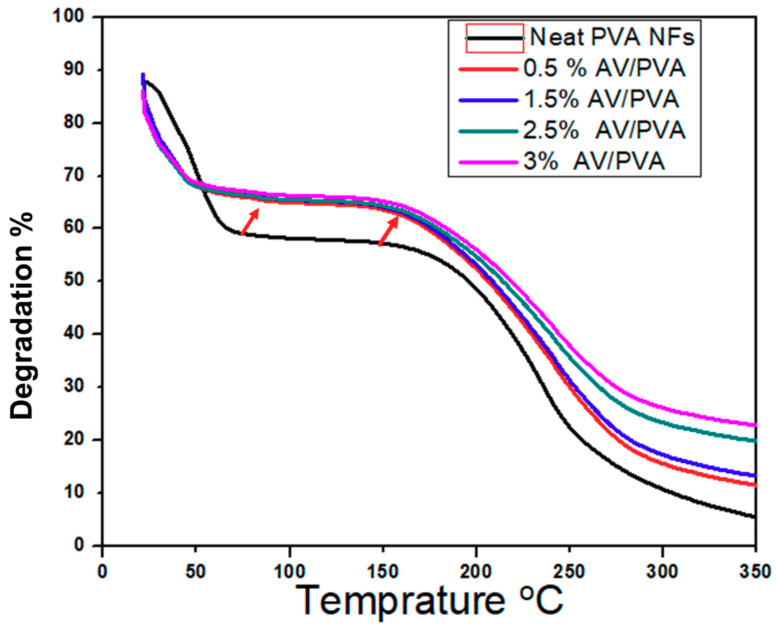
TGA spectra of neat PVA and AV/PVA nanofibers.

In order to investigate the degradation profile of AV/PVA nanofibers, TGA analyss was performed, as shown in the Figure 9. It was confirmed that the thermal stability of the AV/PVA nanofibers was increased by incorporating the AV in the PVA nanofibers. This means the developed product could be used in the high temperature environment such as 80 °C and above. The TGA spectra also confirmed that the residual amount was increased as the concentration of AV was increased, as shown in the Figure 9.

### 3.7. Antimicrobial Activity of AV/PVA Electrospun Nanofibers

**Figure 10:**
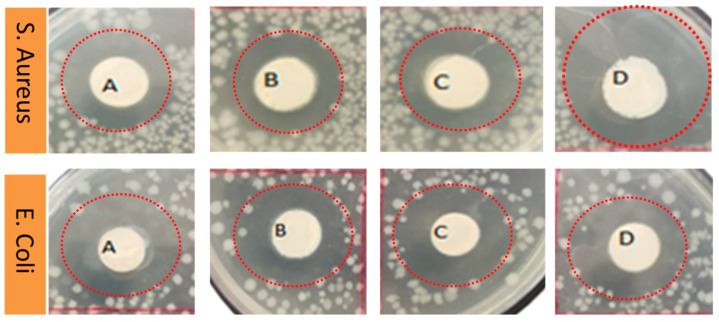
Antimicrobial activity of AV/PVA nanofibers (NFs). (**A**) 0.5% AV/PVA NFs; (**B**) 1.5% AV/PVA NFs; (**C**) 2.5% AV/PVA NFs; (**D**) 3% AV/PVA NFs.

**Figure 11:**
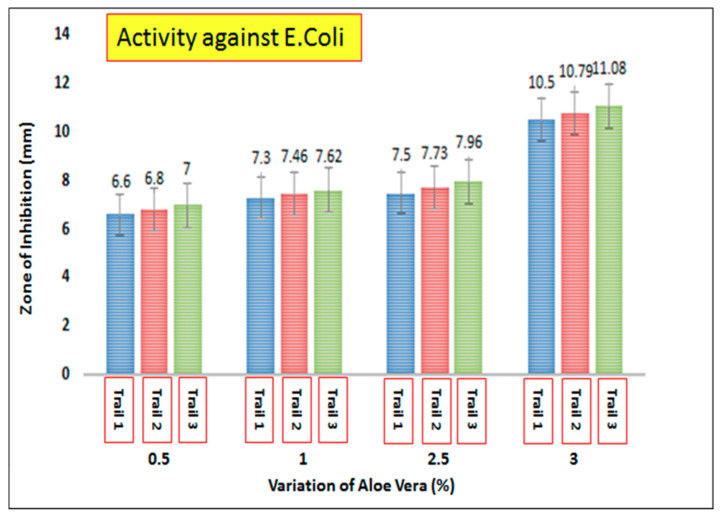
Antibacterial efficiency of AV/PVA nanofibers against *Escherichia coli*.

**Figure 12:**
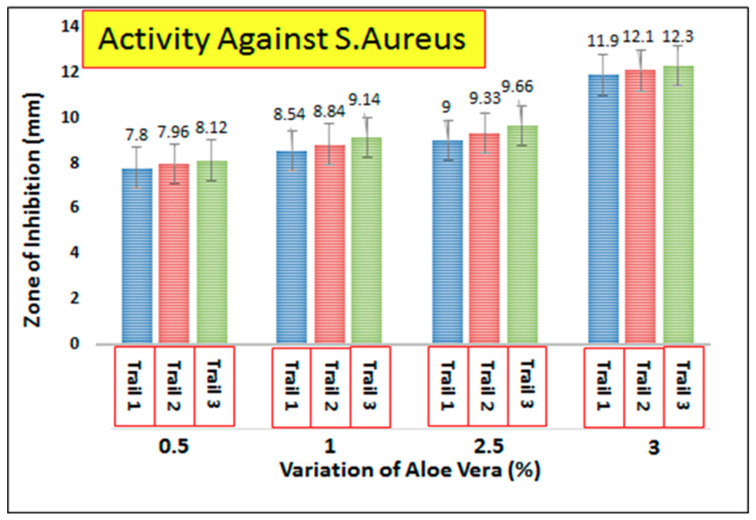
Antibacterial efficiency of AV/PVA nanofibers against *Staphylococcus aureus*.

AV/PVA electrospun nanofibers were screened to determine the antimicrobial activity of the resultant nanofibers. Figure 10 shows the zone of inhibition around the produced samples. It was observed that AV/PVA nanofibers exhibit strong antimicrobial activity against *Staphylococcus aureus* and *Escherichia coli*. The antimicrobial activity is due to anthraquinones, pyrocatechol, isolated from the exudate of Aloe Vera [50]. The cinnamic acid Aloe Vera also takes part in antimicrobial activity by inhibiting glucose uptake in resting bacteria, and slowing down the enzymatic activity of microorganisms [51]. When the specimens were positioned above the bacterial environment, the antimicrobial mediator moves inward from the bacterial cell wall because the surface of bacteria are negatively charged. Therefore, polymer cations are effective for antibacterial activity. After diffusion through the cell wall, the antimicrobial agent causes leakage of the cytoplasmic component from the cytoplasmic membrane and ultimately causes death of the bacteria [52]. Three trials were performed with each concentration of AV/PVA electrospun nanofibers. Figure 11 shows the antibacterial activity against *E. coli* bacteria. It can be seen that at 0.5% concentration of AV the zone of inhibition for Trials 1–3 was calculated as 6.6, 6.8, and 7 mm, respectively. All three trials show antibacterial activity with slight variation in the zone of inhibition. It can also be seen that as the concentration of AV increase, the antibacterial activity of the nanofibers also increases. At 3% higher, antibacterial activity was observed with a zone of inhibition value of 10.5 mm (Trial 1), 10.79 mm (Trial 2), and 11.08 mm (Trial 3). Figure 12 shows the antibacterial activity of AV/PVA electrospun nanofibers against *S. aureus* bacteria. Minimum activity was found at 0.5% AV concentration, with the zone of inhibition 7.8 mm (Trial 1), 7.86 mm (Trial 2), and 8.12 mm (Trial 3). Maximum activity was found at 3% AV concentration, with zone of inhibition 11.9 mm (Trial 1), 12.1 mm (Trial 2), and 12.3 mm (Trial 3). It can also be concluded from the results that nanofibers show excellent antibacterial activity against *S. aureus* bacteria as compare with *E. coli*. The possible reason behind this could be that the cell membrane of *E. coli* is thicker than that of *S. aureus* bacteria [52].

## 4. Conclusions

This study focused on the development of four different types of AV/PVA (0.5%, 1.5%, 2.5%, and 3%) electrospun nanofibers. The SEM analysis confirmed that all the developed fibers were smooth and bead free. At a higher concentration of Aleo vera, finer fibers were obtained. The FTIR analysis of the developed samples confirmed that there was no chemical interaction took place between Aloe Vera and PVA, and as a result no new peak appeared in the spectra. From the FTIR results, it was also concluded that as the concentration of Aleo vera increased, the quantity of OH^−1^ also increased, hence the tendency of the material to absorb moisture increased. The XRD analysis confirmed that by increasing the concentration of Aloe Vera, there was an increase of the amorphous regions in the AV/PVA electrospun nanofibers. The TGA spectra confirmed that the developed web for protective clothing had a suitable profile of thermal stability and a loaded amount of AV. In addition, the ICP study confirmed that AV had a very slow release behavior, which is appreciable for protective clothing. The antimicrobial activity of AV/PVA electrospun nanofibers is excellent, and therefore these fibers are best suited for the preparation of protection clothes (gowns, face masks, etc.) used against COVID-19. From the antimicrobial results, it can also be concluded that AV/PVA shows the highest antimicrobial activity against *S. aureus* as compare with *E. coli* bacteria.
